# Phosphoinositides in plant-pathogen interaction: trends and perspectives

**DOI:** 10.1007/s44154-023-00082-5

**Published:** 2023-04-06

**Authors:** Fauzia Zarreen, Kamal Kumar, Supriya Chakraborty

**Affiliations:** Molecular Virology Laboratory, School of Life Science, Jawaharlal Nehru University, New Delhi, 110067 India

**Keywords:** Plant virus, Kinases, Phosphoinositides, Phosphatidylinositol, Plant-pathogen, Signalling

## Abstract

Phosphoinositides are important regulatory membrane lipids, with a role in plant development and cellular function. Emerging evidence indicates that phosphoinositides play crucial roles in plant defence and are also utilized by pathogens for infection. In this review, we highlight the role of phosphoinositides in plant-pathogen interaction and the implication of this remarkable convergence in the battle against plant diseases.

## Introduction

Lipids derived from Phosphatidylinositol (PtdIns) i.e., phosphoinositides are an important class of cellular phospholipids which play an important role in cell-signalling pathways, actin-cytoskeletal remodelling and membrane dynamics. Further, lipid-protein interaction also forms the basis of phospholipid-based signalling in plant immunity. Here, we discuss phosphoinositides biosynthesis and mode of action, and their key functions in plant defence. This review highlights the significant findings in the field of phosphoinositides in plant-pathogen interactions and offers a fresh perspective on the importance of these macromolecules in the battle against plant diseases.

## Biosynthesis of phosphoinositides in plants

**Fig. 1:**
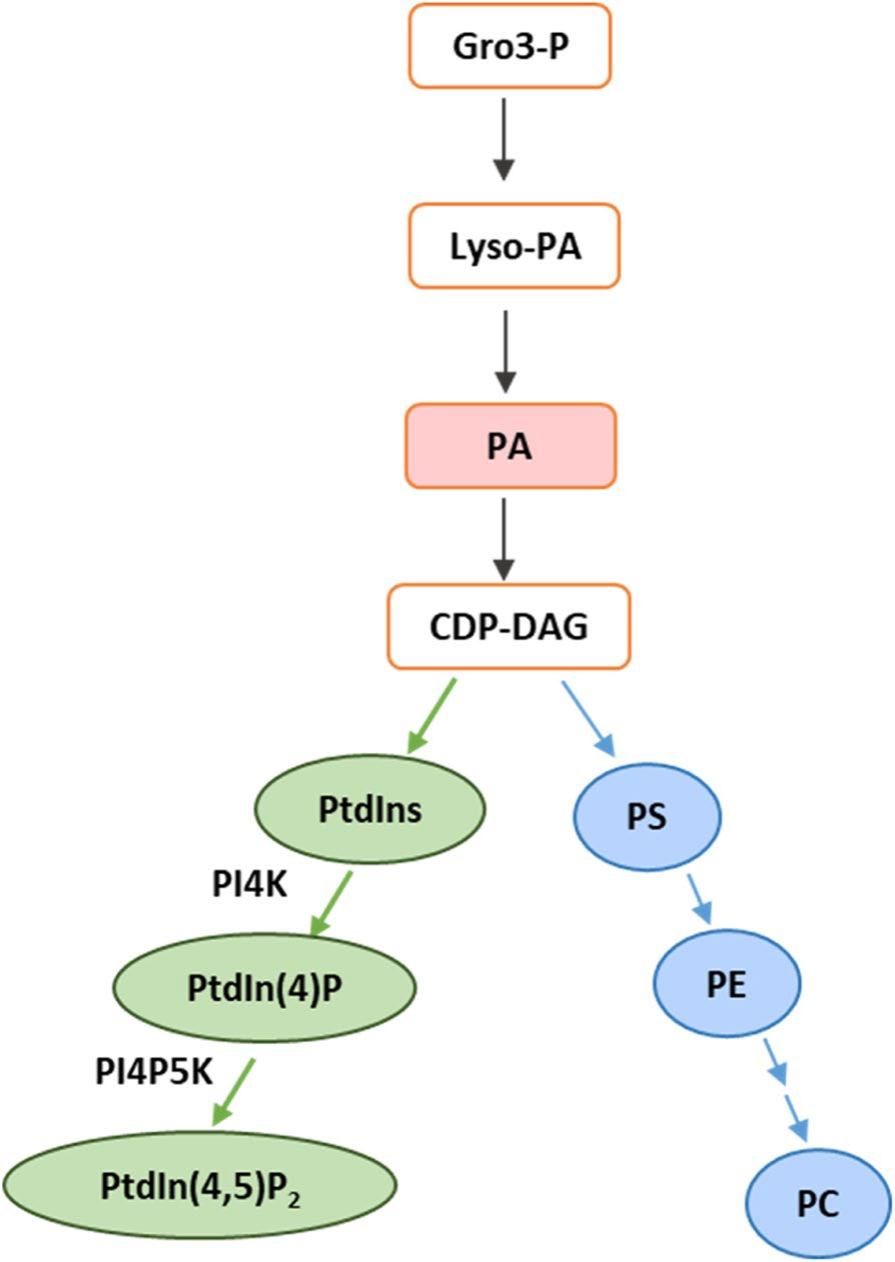
Pathway for the biosynthesis of phosphoinositides. Phosphoinositides are synthesized via the cytidine diphosphate–Diacylglycerol (CDP-DAG) pathway. Gro-3-P, glycerol-3-phosphate; Lyso-PA, lysophosphatidic acid; PA, phosphatidic acid; CDP-DAG, cytidine diphosphate diacylglycerol; PtdIns, phosphatidylinositol; PI4K, Phosphatidylinositol-4 kinase; PI4P or PtdIn(4)P, phosphatidylinositol-4-phosphate; PI4P5K, phosphatidylinositol 4-phosphate 5-kinase; PI(4,5)P_2_ or PtdIn(4,5)P_2_, phosphatidylinositol-4,5-bisphosphate; PS, phosphatidylserine; PE, phosphatidylethanolamine; PC, phosphatidylcholine

In a eukaryotic cell, phosphoinositides are synthesized via the cytidine diphosphate – diacylglycerol (CDP-DAG) pathway. The nucleotide intermediate CDP-DAG reacts with free inositol to form PtdIns in the endoplasmic reticulum (ER) (Fig. 1). Phosphorylated derivatives of PtdIns are then produced by a set of phosphatidylinositol kinases (PIKs) and phosphatidylinositol phosphatases. PIKs phosphorylate the -hydroxyl (OH) group of inositol ring at positions 3′, 4′, and 5′ to generate seven phosphatidylinositol phosphates (PtdInsPs or PIPs) – three monophosphates [PtdIn(3) P, PtdIn(4) P and PtdIn(5)P], three bisphosphates [PtdIn(3,4)P_2_, PtdIn(4,5)P_2_ and PtdIn(3,5)P_2_] and one triphosphate [PtdIn(3,4,5)P_3_]. However, in plants, only five of the seven PIPs have been detected. The presence of PtdIn(3,4)P_2_ and PtdIn(3,4,5)P_3_ has not been confirmed in plants. PtdIn(4) P is the most abundant in the plant cell (Munnik and Vermeer 2010).

In plants, PtdIn(3) P and PtdIn(4) P are formed from PtdIns by the action of specific kinases: the PI3-kinase (VPS34) and 2 of PI4-kinases, namely PI4Kα, PI4Kβ (Mueller-Roeber and Pical 2002). Twelve isoforms of PI4K have been identified in Arabidopsis, which can be divided into two subfamilies, type II (PI4Kγ 1–8) and type III (PI4K α1, α2, β1 and β2) (Mueller-Roeber and Pical, 2002). The type III PI4Ks members contain a PI3K/PI4K catalytic domain and contain PtdIns-binding domains such as Pleckstrin Homology (PH) domain (in PI4Kα) or PI4K charged (PPC) domain (in PI4Kβ) (Mueller-Roeber and Pical, 2002; Stevenson-Paulik et al., 2003, Fig. 2). Further, type II PI4Ks contain PI3/4 kinase domain but lack a PtdIns-binding domain and have not been shown to synthesize PtdIn(4) P via a PtdIns-catalyzed pathway (Galvão et al., 2008). However, type II PI4Ks have been shown to phosphorylate various other proteins and only a few reports of the physiological function exist (Tang et al., 2016; Galvão et al., 2008; Liu et al., 2013; Alves-Ferreira et al., 2007). For detail on PI4Ks, we refer the readers to Szumlanski and Nielsen (2009) and Mueller-Roeber and Pical (2002). While PI3K and PI4Ks have been identified in plants, an enzyme catalyzing the generation of PtdIn(5) P directly from PtdIns has not been identified. Further, PtdIns can be dephosphorylated by phosphatases and phospholipases. Although the *A. thaliana* genome encodes several PtdIn phosphatases and phospholipases, only a few of these have been assigned particular catalytic or physiological functions. Important families of PtdIn phosphatases include Suppressor of Actin (SAC) phosphatases which hydrolyse phosphate group of phosphoinositides at multiple positions (Zhong and Ye, 2003), Phosphatase and Tensin homolog deleted on chromosome 10 (PTEN) related enzyme that dephosphorylates PtdIn(3) P, PtdIn(3,4)P_2_, and PtdIn(3,5)P_2_ (Pribat et al., 2012) and PtdIn-5 phosphatase (5PTase) family which hydrolyses PtdIn(4,5)P_2_ (Gunesekera et al., 2007). Further, PtdIn can also be cleaved by PtdIn-specific phospholipase C (PI-PLC) (Chen et al., 2011). The *A. thaliana* genome encodes nine isoforms of PI-PLCs (Mueller-Roeber and Pical, 2002) which are activated by Ca^2+^ (Pokotylo et al., 2013).

**Fig. 2:**
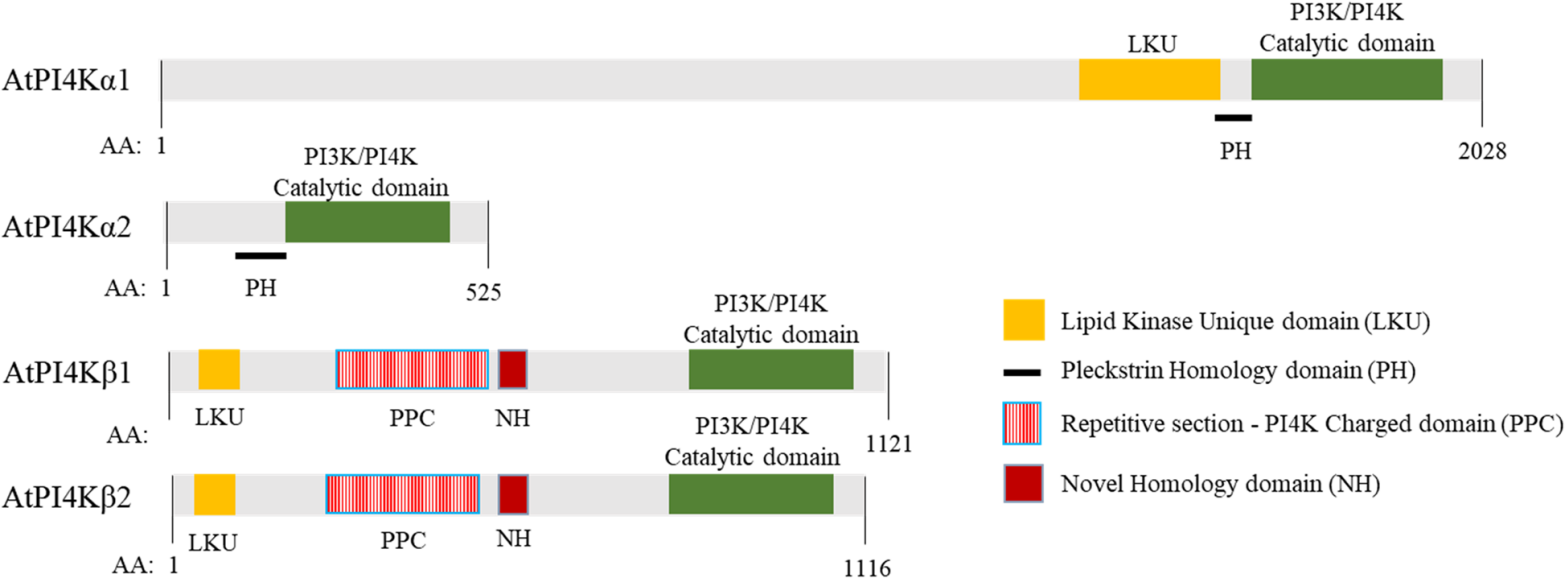
Domain architectures and multiple sequence alignment of *Arabidopsis thaliana* phosphatidylinositol 4- kinases. PI4Ks are characterized by a predicted catalytic domain, and are divided into three groups: alpha (α), beta (β), and gamma (γ), based on additional domains. The α and β groups, with the exception of AtPI4Kα2, have a lipid kinase unique (LKU) domain or the helical domain. The β group members have a novel homology (NH) domain, and also contain an N-terminal LKU domain. Both α and β groups contain a PtdIns-binding domain, Pleckstrin Homology (PH) and Pi4K charged (PPC) domain, respectively

## Dynamics of phosphoinositides biosynthesis and distribution

PIKs and PtdIn phosphatases are responsible for the rapid and dynamic turnover and distribution of phosphoinositides (Reviewed Heilmann 2016a). The PtdIn pathway enzymes exhibit different patterns of expression, with some being expressed ubiquitously, whereas others are restricted to certain developmental stages or cell types (Heilmann, 2016b). Spatio-temporal dynamics of PtdIn change rapidly in response to various developmental and environmental cues. For instance, changes in PtdIn level have been observed in response to osmotic stress (DeWald et al., 2001; Einspahr et al., 1988a, b; Heilmann et al., 1999, 2001; König et al., 2007, 2008), heat (Mishkind et al., 2009), nutritional deficiency, developmental cues such as gravitropic curvature (Perera et al., 1999, 2001), ER stress response (Kanehara et al., 2015), and pollen tube growth (Bloch et al., 2016) (Table 1). Further, biotic stress including haustoria biogenesis (Qin et al., 2020), bacterial as well as a defence response to the fungal pathogen (Antignani et al., 2015; Qin et al., 2020) also results in changes in the PtdIn levels (Table 2). The changes in the PtdIn levels are generally associated with membrane deformation, endomembrane organization, cytoskeletal rearrangement and changes in membrane trafficking Gerth et al. 2017b; Heilmann 2016a, b; Ischebeck et al. 2008, 2011, 2013; Noack and Jaillais, 2017; Sousa et al., 2008; Tejos et al., 2014; Thole and Nielsen, 2008 König et al., 2008; Zhao et al., 2010; Mei et al., 2012, Stenzel et al., 2008, Munnik and Nielsen, 2011; Heilmann and Heilmann, 2013; Heilmann and Ischebeck, 2016).

**Table 1:** Role of phosphoinositides in abiotic stress

Stress	Phosphoinositides /Enzyme	Plant/Site of expression	Role	References
Salinity and Osmotic Stress	PI (4,5) P_2_ Phosphatidylinositol 4,5-bisphosphate	Root, Leaf	Increases concentration of IP_3_ and mobilisation of Ca^2+^ ions into cytosol from ER and vacuoles	Pical et al., 1999; DeWald et al., 2001; Munnik et al., 2014; Morales et al., 2019
	PLC Phospholipase C	Rice (OsPLC1) Plasma membrane	Hydrolyses Phosphatidylinositol 4-phosphate to elicit Ca^2+^ ions and provide salt tolerance	Li et al., 2017; Morales et al., 2019
	PI4K	*Arabidopsis thaliana* (AtPI4Kγ3) Nucleus	Upregulated in salt stress and decreases the accumulation of ROS to provide plant tolerance	Akhter et al., 2016; Morales et al., 2019
	PLC	AtPLC1 Root, Stem, Leaf	Upregulated in dehydration and salt stress to provide plant tolerance	Hirayama et al., 1995; Morales et al., 2019
	PLC	Wheat TaPLC1 Root, Stem, Old leaves	Upregulated during seed germination and seedling growth to provide salt and drought tolerance	Zhang et al., 2014
	Phosphatidylinositol bisphosphate (PIP2) and Phosphatidic acid (PA)	Rice leaves	Upregulated in salt stress	Darwish et al., 2009
	Phosphatidylinositol 5-monophosphate (PI5P)	*Vicia sativa and Chlamydomonas*	Increased levels under hyperosmotic stress	Meijer et al., 2001
	PI3P, PI4P and PI5P	–	Increased levels during hyperosmotic stress	Meijer et al., 2001; Konig et al., 2007; Hou et al., 2016
	PI(4,5)P_2_* and PI4P* InsP_6_#, DAG# and PLC#	*Dunalliela salina*	Breakdown (*) Increased (#) thereby reducing the effects of high salt stress	Ha and Thompson Jr., 1991; Cote and Crain, 1993
	IP_3_	*Lamprothamnium succinctum* (green alga)	Increased production during hypo-osmotic stress	Cote and Crain, 1993
	PI(3,5)P_2_	*Saccharomyces cerevisiae*	Increased levels during hyperosmotic stress	Dove et al., 1997; Meijer et al., 1999
Drought	PI specific PLC	Maize (ZmPLC1) and Tobacco	Upregulated and maintains the stability of PtdIns that results in drought tolerance	Zhai et al., 2013; Morales et al., 2019
	PLC	*Torenia fournieri* (TfPLC2) Leaves and Stem	Upregulated and results in drought tolerance	Song and Han, 2008; Morales et al., 2019
	PLC	Wheat TaPLC1 Root, Stem and old leaves	Upregulated during seed germination and seedling growth to provide salt and drought tolerance	Zhang et al., 2014
	InsP3 (IP_3_)	*A. thaliana*	Altered level leads to drought tolerance	Perera et al., 2008; Hou et al., 2016
	PLD, AtPLD δ, LePLD β1	*Vigna unguiculata, Craterostigma* *plantagineum. A. thaliana*, Tomato	Increased levels	Maarouf et al., 1999; Frank et al., 2000; Katagiri et al., 2001; Laxalt et al., 2001
Heat	PI(4,5)P_2_	BY-2 tobacco cells	Overexpression results in tolerance to heat	Van Leeuwan et al., 2007; Ndamukong et al., 2010; Morales et al., 2019
Nutrient deprivation (starvation of sucrose)	PI(4,5)P_2_	*A. thaliana*	Increased levels upon starvation of sucrose	Contento et al., 2004; Morales et al., 2019
Oxidative	PLD	*A. thaliana*	Increased levels upon oxidative stress	Sang et al., 2001

**Table 2:** Role of phosphoinositides in biotic stress

Pathogen	Species	PI/Enzyme	Plant/Site of expression (action)	Role	References
Fungi	*Sclerotinia sclerotiorum* *Magneporthe grisea* *Mycosphaerella pinodes*	PLC2 PLC1 PI (4,5) P_2_ and Ins (1,4,5) P_3_	Transgenic *B napus,* Rice, Pea (epicotyls)	Infection reduces with increased expression	Toyoda et al., 1993; Song and Goodman, 2002; Nokhrina et al., 2014; Morales et al., 2019
Bacteria	*Xanthomonas oryzae* pv. *oryzae* *Pseudomonas syringae*	PLD Ins (1,4,5) P_3_	suspension cells	Levels reduced during infection	Young et al., 1996; Shigaki and Bhattacharyya, 2000.
Virus	*Tomato bushy stunt virus* *Chilli leaf curl virus*	PI3P PI4K	*N. benthamiana*	Positively regulates virus replication	Feng et al., 2019, Mansi et al., 2019

The mechanism underlying the dynamic turnover of PtdIn under various environmental cues remains unclear. It is interesting to note that no significant change in the transcription of genes encoding enzymes for PtdIn metabolism was observed upon challenge by a wide range of stress (Zimmermann et al., 2004; Heilmann, 2016b). However, transcription of some genes such as PLC1 & PIP5K1 is induced by osmotic stress and exogenous auxin application respectively in *A. thaliana* (Hirayama et al., 1995, Tejos et al., 2014). Some studies indicate post-translational modifications of PtdIn-catalysing enzymes in response to stress (Westergren et al., 2001). For example, phosphorylation of PIP5K6 by mitogen-activated protein kinase (MAPK) MPK6 upon PAMP (Pathogen-associated molecular patterns) perception reduces the activity of PIP5K6 kinases (Menzel et al., 2019). Further, PIP5K6 expression on PIP5K6 protein abundance largely remained unchanged upon PAMP perception.

## Modes of phosphoinositides action

In the cell, PtdIn function either as intact or hydrolyzed phosphoinositides. Intact PtdIn is found predominantly at the cytosolic face of the cellular membrane and acts as ligand for partner proteins and can influence the biophysical properties of the plasma membrane (PM, Falkenburger et al., 2010). The characteristic head groups of PtdIn with distinct phosphorylation patterns protrude into the cytosol and can bind to partner proteins containing specific phosphoinositides-recognition domains (Takenawa, 2010) such as Pleckstrin homology (PH) domains, Fab1 YOTB Vac1 EEA1 (FYVE) domains and phagocytic oxidase (PX) domains (Lemmon, 2003). The binding of a PtdIn ligand to a protein could affect the activity or function of the target protein and can result in the recruitment of a particular protein to membrane areas enriched in particular (spatial distribution) phosphoinositides. However, only some proteins having PtdIn recognition domains display a high degree of specificity for particular phosphoinositides. For example, during endocytosis, epsin NH_2_-terminal homology (ENTH) domain-containing proteins bind preferentially to PtdIn(4,5)P_2_ (Itoh & Takenawa, 2004), other regulatory proteins prefer PtdIn(4) P, while most PH domains do not exhibit such preferences and can bind to any phosphoinositides. A recent study has identified PtdIn(4)P as the main PtdIn required to establish the surface charge on the plasma membrane (Simon et al., 2016). Further, phosphatidylserine (PS) in combination with PtdIn(4)P organizes the intracellular electrostatic gradient along the endocytic pathway (Platre et al., 2018). This change in the biophysical property of the membrane controls the localization and function of many proteins. In the case of plant development, PtdIn can act as a recruiter to facilitate auxin transport. For example, D6 protein kinase (D6PK), a serine/threonine kinase which helps in PIN-mediated auxin transport, binds to both phosphoinositide and phosphatidic acid through its domain K-rich motif. Further, this binding helps to recruit D6PK towards the plasma membrane to maintain directionality in a polar manner (Barbosa et al., 2016). Another report highlighted the role of phospholipids in endomembrane reticulum (ER) - PM contact and cell-to-cell signalling. Here, MCTP (Multiple C2 domain and Transmembrane region Protein), a plasmodesmata protein binds to both ER and PM by a transmembrane region and anionic phospholipid. This enables MCTPs to respond in cell-to-cell signalling by changing to their different conformations (Brault et al., 2019). In another example, the vesicle tethering complex exocysts subunit EXO70A1 interacts with PtdIn(4)P and phosphatidic acid and recruits the exocyst to the PM, thereby driving plant cell polarity and morphogenesis (Synek et al., 2021). Further, plant-specific membrane protein REMORIN (REMs) anchors itself to the PM by its specific interaction with PtdIn(4)P (Gronnier et al., 2017).

Further, hydrolyzed PtdIns could serve as a precursor for further downstream signalling. The hydrolysis of PtdIn(4,5)P_2_ by PLC yields DAG and Inositol 1,4,5-triphosphate (IP_3_)_._ Unlike the mammalian system, a signalling function for DAG in plants has not been reported. In plants, DAG is rapidly phosphorylated to phosphatidic acid (PA), which has been shown to have several regulating functions. Similarly, the role of soluble IP_3_ is only beginning to be uncovered in plants.

### Role of phosphoinositides in regulating plant nuclear function

PtdIns are important regulators of cellular and physiological processes in eukaryotes. In addition to PtdIn and PtdIn-pathway enzymes in PM, their presence in the nucleus was also reported in animals, plants and yeast. The fate of the nuclear PtdIn pathway is generally governed by nuclear kinases and phosphatases rather than PM-bound PtdIn-enzymes, illustrating the novel intra-nuclear signalling pathway composed of nuclear-generated PtdIn. It is well established that the nuclear PtdIns regulate many processes including nuclear size, nuclear membrane reassembly, chromatin structure, DNA replication, localization of transcription factors, transcription, RNA splicing, mRNA export, and cell cycle progression in animal and yeast cells.

In plants, the role of PtdIns in membrane trafficking events and endomembrane organization and cytoskeletal rearrangement is well documented, however, their critical signalling role in nuclear functions is only being discovered. A few PtdIns pathway enzymes or enzyme activities and their products also have been found in the nucleus of plants. The *A. thaliana* PIP5K isoforms, 4, 5, 6, 9, and 11 contain possible nuclear localization sequence (NLS) sequences (by WoLF PSORT, Horton et al., 2007). GFP-tagged AtPIP5K isoform 9 has been shown to localize to both the nucleus and the PM (Lou et al., 2007). Other PtdIn pathway enzymes reported in plant nuclei include myo-inositol phosphate synthase (MIPS), PI3K, PI4K, PI4P5K, PI-PLC, Diacylglycerolkinase (DAGK) and Inositol 5-phosphatase (Dieck et al., 2012a, b). Further, the presence of phospholipids including phosphoinositides, phosphatidylserine (PS), phosphatidylethanolamine (PE) and phosphatidylcholine (PC) in plant nuclei has been confirmed (Philipp et al., 1976). Lipid profiles of *Nicotiana tabacum* cells indicate that PIs account for a higher molecular percentage of total lipids compared to the protoplast PM lipids (Dieck et al., 2012a, b). In another study Gerth et al. (2017a), characterize the four putative NLSs present in Arabidopsis PIP5K2. Mutation in the basic residue of NLS precludes AtPIP5K2 from importing into the nucleus. AtPIP5K2 also interacts with selected alpha-importin isoforms, an important component of nuclear-import machinery. Further, the immunofluorescence experiment confirmed the presence of AtPIP5K2 substrate, PtdIn(4)P and the reaction product PtdIn(4,5)P_2_ in the plant nucleus (Gerth et al., 2017a). These experiments indicate that PtdIns can be synthesised in the plant nuclei using nuclear-import of enzymes of the PtdIns pathway.

Changes in PtdIn levels during the cell cycle have been documented in plants. Induction of somatic embryogenesis in coffee plants causes a transient change in levels of PtdIn(3)P and PtdIn(4)P (Ek-Ramos et al., 2003). In a recent study, Von der Mark et al. (2022) show that the exclusion of PIP5K1 from the nucleus disrupts xylem differentiation (Von der Mark et al., 2022). Further, the expression of the human phosphatidylinositol (4)-phosphate 5-kinase (HsPIP5K) 1α in *N. tabacum* cells resulted in an increase in PtdIn(4,5)P_2_ levels in their isolated nuclei, which led to altered nuclear lipids and nuclear functions (Dieck et al., 2012a, b). The increase in PtdIn(4,5)P_2_ levels resulted in a significant reduction in DNA replication, histone 3 lysine 9 acetylation (H3K9ac) and phosphorylation of the retinoblastoma protein (pRb) (Dieck et al., 2012a, b). An increase in PtdIn(4,5)P_2_ in the nucleus of the transformed *N. tabacum* cells could have inhibited the DNA polymerase activity and/or Topoisomerase activity, thus slowing down DNA polymerase progression. A direct binding of human Topoisomerase IIα to PtdIn(4,5)P_2_ and the consequently decreased topoisomerase activity have already been reported (Lewis et al., 2011). Additionally, studies have shown PtdIns to decrease α, δ, and ε DNA polymerase activity in animals (Shoji-Kawaguchi et al., 1995). In animals, chromatin remodelling proteins are sensitive to the PtdIns pathway lipid. In one such example in plants, exogenously added PtdIn(5)P causes relocalization of histone trimethytransferase ATX1 from the nucleus to the PM and subcellular vesicles resulting in a decrease in ATX1-dependent H3K4 trimethylation levels (Alvarez-Venegas et al., 2003). Cross-talk between H3K4 trimethylation is known to enhance H3K9 acetylation in plants, hence, it is plausible that perturbation of the PtdIn(4,5)P_2_ levels in the cell could also alter H3K9 acetylation. Further, a role for PtdIns in regulating transcription and RNA processing has been described in the animal system. For example, PtdIn(4,5)P_2_ decreased the inhibition of RNA polymerase activity by H1 (Yu et al., 1998) and mutation in the nuclear PIP5K skittles in *D. melanogaster* lead to a decrease in transcriptionally active chromatin (Cheng & Shearn, 2004). Although the exogenous addition of PtdIn(4)P and PtdIn(5)P causes differential gene expression in *A. thaliana,* the role of plant nuclear phosphoinositides pathway in transcription and RNA processing remains to be described.

## Role of phosphoinositides in plant-pathogen interaction

Many lipids and lipid-related compounds are known to have a role in plant defence signalling and responses to biotrophic and necrotrophic diseases. However, our knowledge of the possible roles of phosphoinositide in plant defence is limited. The role of PtdIns in biotic stress is rapidly being uncovered in bacterial, fungal and viral infections. Here, we discuss the current understanding of the direct and indirect role of PtdIns in plant immunity and various stages of pathogen infection.

### Plant defence signalling

The transient over-expression of tomato PLCs in *N. benthamiana* enhances the biotrophic fungal pathogen *Cladosporium fulvum* effector protein, Avr4-triggered hypersensitive response (Abd-El-Haliem et al., 2016). Expression of *Oryza sativa* specific *PI-PLC1*(*OsPI-PLC1*) was induced by various chemical and biological inducers of the plant defence pathway including salicylic acid (SA), benzo-(1,2,3)-thiadiazole-7-carbothioic (BTH), an SA analogue, jasmonic acid (JA), *Pseudomonas syringae* pv *syringae* and wounding (Song & Goodman, 2002). Perception of the pathogen-associated molecular pattern (PAMP), flg22, by the pattern-recognition receptor, FLAGELLIN SENSITIVE 2 (FLS2) at the surface of *A. thaliana* cells, led to a decrease in the levels and reduced plasma membrane association of PtdIn(4,5)P_2_ (Menzel et al., 2019). In mesophyll protoplasts, flg22 triggers the phosphorylation of phosphatidylinositol 4-phosphate 5-kinase (PI4P5K) by MPK6 resulting in reduced PtdIn(4,5)P_2_ production and reduced catalytic activity of PI4P5K. Mutation of the MPK6-targeted residues or protoplasts from *mpk6* mutants does not affect the catalytic activity of PI4P5K (Menzel et al., 2019). Further, this PI4P5K expression and protein abundance were very slightly affected by the flg22 treatment, indicating that PI4P5K activity is controlled post-translationally (Menzel et al., 2019). In another example, the recognition of two *P. syringae* Avr proteins, AvrRpm1 and AvrRpt2, causes biphasic accumulation of PA before cell death in Arabidopsis (Andersson et al., 2006). This increase in PA is mediated by PI-PLC and PLD. PI-PLC hydrolyses PtdIn(4)P and PtdIn(4,5)P_2_ to produce monophosphatidylinositol, inositol di-phosphate(IP_2_) and IP_3_ and subsequent influx of extracellular Ca^2+^ and in concert with DAGK produces PA. Interestingly, inhibition of phospholipase reduced PA accumulation and the severity of the hypersensitive response (HR, Andersson et al., 2006).

**Fig. 3:**
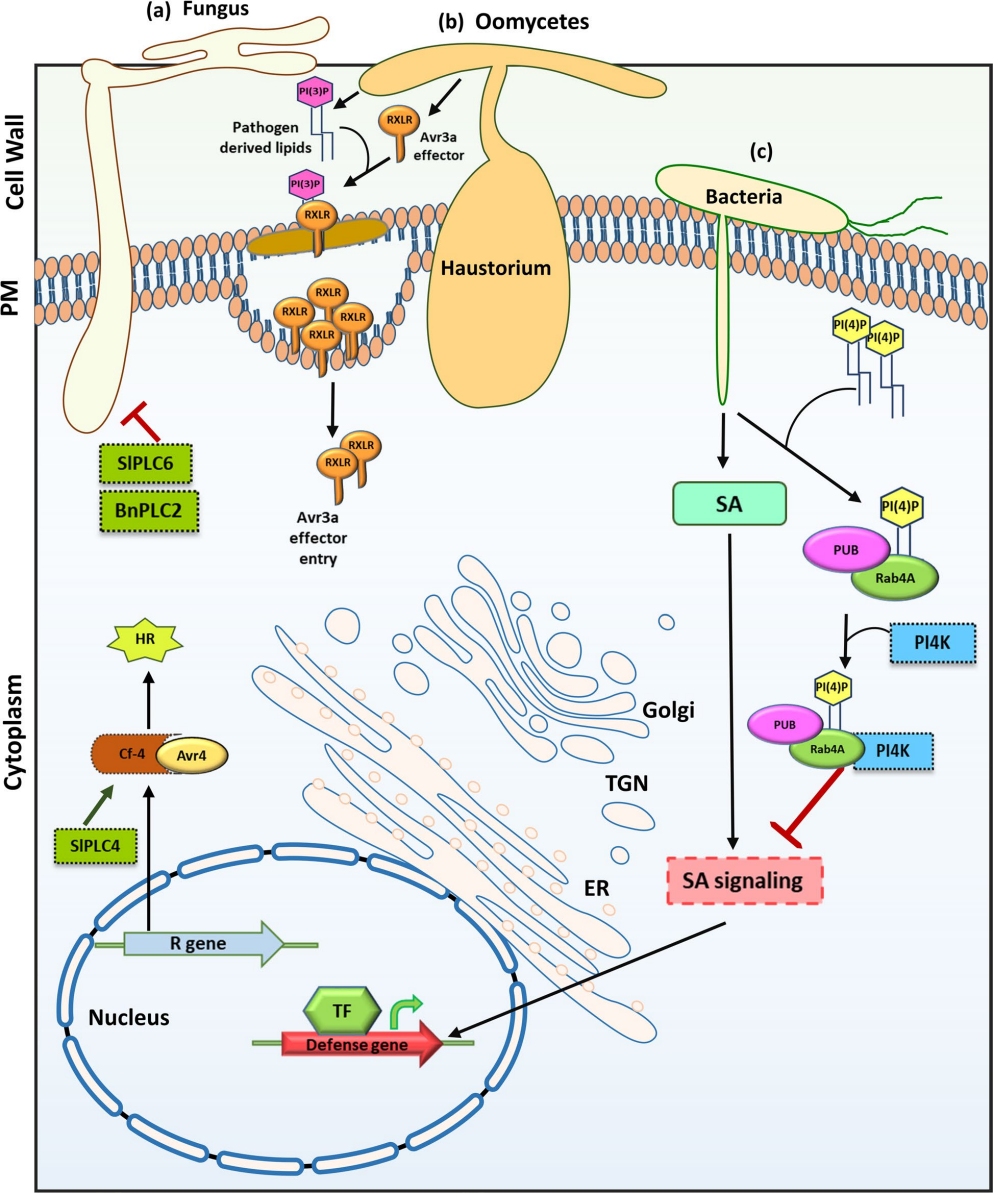
Role of phosphoinositides in fungal, oomycetes and bacterial infection in plants. **a** *Brassica napus* phospholipase C2 (BnPLC2) and *Solanum lycopersicum* PLC4 (SlPLC4) negatively regulate fungal infection. *Solanum lycopersicum* PLC6 (SlPLC6) positively regulates effector Avr4/Cf-4 (R gene) induced HR. **b** Phosphatidylinositol-3-phosphate (PI(3)P) mediates the entry of oomycete effector Avr3a into the host cell. **c** In bacterial infection, PUB13, a Plant U-Box (PUB) family of E3 ligase proteins and a negative regulator of SAdependent defense responses, interacts with Rab effector proteins (RabA4B) and PI(4)P and then recruits phosphatidylinositol 4- kinase (PI4K) to repress SA-dependent gene expression and defense response. BnPLC2, *Brassica napus* phospholipase C2; SlPLC4, *Solanum lycopersicum* PLC4; SlPLC6, *Solanum lycopersicum*; PI(3)P, phosphatidylinositol-3-phosphate; PUB, Plant U-Box; PI(4)P, phosphatidylinositol-4-phosphate; PI4K, phosphatidylinositol 4- kinase; Rab, Ras-related protein; HR, hypersensitive response; SA; salicylic acid; PM; plasma membrane; ER, endoplasmic reticulum; TGN, *trans*-golgi network; TF, transcription factor

The application of fungal elicitor xylanase in tomato causes accumulation of PtdIn(4)P in the extracellular medium in a rapid, dose- and time-dependent manner and the exogenous application of PtdIn(4)P mimics xylanase effects, suggesting its putative role as an intercellular signalling molecule (Gonorazky et al., 2008). Transgenic *Brassica napus* overexpressing phospholipase C2 (BnPLC2) showed enhanced tolerance to the fungus *Sclerotinia sclerotiorum* (Nokhrina et al., 2014, Fig. 3). *Solanum lycopersicum* PLC, *SlPLC4* and *SlPLC6* were differentially regulated in response to infection with the pathogenic fungus *Cladosporium fulvum* in *C. fulvum-*resistant Cf-4 tomato (Vossen et al., 2010)*.* Silencing of *SlPLC4* impaired the *C. fulvum* effector Avr4/Cf-4 (R gene) induced HR and resulted in increased colonization of Cf-4 plants by *C. fulvum*. However, silencing of *SlPLC6* did not affect HR but caused increased colonization by the fungus (Vossen et al., 2010, Fig. 3). Interestingly, resistance to another fungus *Verticillium dahlia* and bacteria *P. syringae* was mediated only by *SlPLC6*, indicating the differential requirement of PLC isoforms for the plant immune response (Vossen et al., 2010). PtdIns have also been shown to play a role in SA-dependent defence responses in *A. thaliana*. PUB13, a Plant U-Box (PUB) family of E3 ligase proteins and a negative regulator of SA-dependent defence responses, interact with Rab effector proteins (RabA4B) through N-terminal domains and with PtdIn(4)P through a C-terminal armadillo domain. RabA4B then recruits the closely related PI4K, PI4Kβ1 and PI4Kβ2 to repress SA-dependent gene expression and defence response (Fig. 3). Mutations in PUB13 and PI4Kβ1/PI4Kβ2 result inactivation of the SA-dependent defence gene and enhanced resistance against *P. syringae* pathovar tomato (Pst) DC3000 (Antignani et al., 2015).

### Pathogen/effector entry

Besides mediating defence signalling, PtdIns play a role in pathogen effector entry into the host cell. Oomycete RXLR-dEER host-targeting signals, and similar signals in fungal effectors, bind to host cell surface PtdIn(3)P and mediate the entry of the effector into the host cell (Kale et al., 2010). PtdIn(3)P binding proteins and exogenous inositol phosphates inhibited the entry of the effector into the cell (Kale et al., 2010, Fig. 3). Further, the *Pseudomonas sojae* effectors Avr1b, Avh5, and Avh331 were reported to bind PtdIn(3)P, and binding required an intact RxLR motif (Kale et al., 2010). The effector domain of *Phytophthora capsici* effector protein AVR3a4 contains a conserved, positively charged patch and this region, rather than the RXLR domain, is required for binding to PtdIns in vitro (Yaeno et al., 2011). Mutations in the positively charged patch of AVR3a4 do not prevent AVR3a from being recognized by the resistance protein R3a, but they do diminish R3a’s capacity to inhibit INF1-induced cell death in plants (Yaeno et al., 2011). Since the level of PtdIn(3) is low in plants, *Phytophthora* species produce PtdIn(3)P to promote the entry of RxLR effectors into host cells (Lu et al., 2013). The accumulation of pathogen-derived PtdIn(3)P was detected using PtdIn(3)P-specific GFP biosensors, which could bind to *P. parasitica* and *P. sojae* hyphae during infection of *N. benthamiana* leaves transiently secreting the biosensors (Lu et al., 2013). The pathogen’s virulence on soybean was decreased by either silencing the PI3K genes, the treatment with LY294002, or the production of PtdIn(3)P-binding proteins, demonstrating that pathogen-synthesized PtdIn(3)P was necessary for maximal virulence. *N. benthamiana* leaves secreting PtdIn(3)P-binding proteins or a PI3P5K considerably enhanced resistance to infection by *P. parasitica* and *P. capsici* (Lu et al., 2013).

### Colonization and hyphal growth

Lipid reporters have been used to study the role of PtdIns in polar tip growth in plants. For example, fluorescent reporters indicate that PtdIn(4,5)P_2_ accumulates in the apical region of the pollen tubes and root hair (Dowd et al., 2006; Tejos et al., 2014; Ischebeck et al., 2013; Helling et al., 2006; Kusano et al., 2008; Sousa et al., 2008; Stenzel et al., 2008; Ischebeck et al., 2011). A similar distribution of PtdIns has been reported during the development of intracellular branched hyphae or arbuscule of the arbuscular mycorrhizal (AM) fungi within root cortical cells of *Medicago truncatula* roots during symbiosis with *Rhizophagus irregularis,* AM fungi. PtdIn(4)P reporters accumulated all along on the periarbuscular membrane (PAM), while PtdIn(4,5)P_2_ reporters show accumulation at small, discrete patches on the PAM on arbuscule trunk (Ivanov and Harrison, 2019), reflecting changes in endomembrane secretion and activity.

In another example of rapid polarized membrane expansion, PtdIn(4,5)P_2_ accumulation was enriched in the extra-invasive hyphal membrane (EIHM) of the hemibiotrophic fungus *Colletotrichum higginsianum* (Ch) in Arabidopsis. Further, PIP5K, enzymes which catalyze PtdIn(4,5)P_2_ production also enriched at the EIHM (Shimada et al., 2019), Interestingly extra haustorial membrane (EHM) of biotrophic powdery mildew fungus did not accumulate PtdIn(4,5)P_2_. In contrast EHM of oomycete *Hyaloperonospora arabidopsis*, the causal pathogen of downy mildew was enriched with PtdIn(4)P indicating that PtdIn content of plant-pathogen interfacial membrane is specific for a pathosystem (Shimada et al., 2019). In contrast, Raushe et al. (2021), have identified a *Solanum tuberosum* phosphoinositide 5-phosphatase (StIPP) which specifically dephosphorylates PtdIn(4,5)P_2_. Upon *Phytophthora infestans* infection, StIPP relocalizes from PM to EHM and may antagonize PtdIn(4,5) P2-mediated plant immunity at the infection site (Raushe et al., 2021).

### Intracellular movement and transmission

Lipids and proteins in the plasma membrane dynamically assemble into membrane domains giving rise to fluid molecular patchwork (Gronnier et al., 2017, Jaillais and Ott, 2020). Emerging evidence suggests that these “nanodomains” provide a dedicated biochemical and biophysical environment for cell-signalling (Gronnier et al., 2019; Jaillais and Ott, 2020). In plants, members of REMORIN (REM) protein families have been characterized as membrane markers for these “nanodomains” (Raffaele et al., 2007, 2009; Jarsch et al., 2014). The physiological functions of REMs have largely been reported in plant-virus interaction and hormone crosstalk. For example, in *Solanaceae*, REM organizes into a cytosolic domain of ~ 70 nm in PM and is also present in the plasmodesmata. Further, REM directly interacts with *Potato virus* X (PVX) movement protein TRIPLE GENE BLOCK PROTEIN 1 (TGBP1) and limits PVX cell-to-cell movement without affecting virus replication (Raffaele et al., 2007 and 2009, Fig. 4). In another example, the loss of function mutant of Arabidopsis REMORIN group 4 (AtREM4) shows reduced susceptibility to geminivirus *Beet Curly Top Virus* (BCTV) and *Beet Severe Curly Top Virus* (BSCTV) (Son et al., 2014). REMs have been shown to promote susceptibility to *P. infestans* (Bozkurt TO et al., 2014) and also play a positive role during the nodulation process in *M truncatula* (Lefebvre et al., 2010). In a recent study, Gronnier et al., (2022) showed that perception of an endogenous peptide hormone RAPID ALKALANIZATION FACTOR 23 (RALF23) by PM receptor kinase FERONIA (FER) and leucine rich repeat extension protein (LRXs) actively modulates PM nanoscale organization of other ligand-binding receptor kinase and inhibits immune signalling (Gronnier et al., 2022). To fulfil these functions, REM needs to localize to the PM and they achieve PM anchoring by interacting with PtdIn(4)P through the C-terminal domain of REM called REM-CA (REM C-terminal anchor) (Gronnier et al., 2017). Interestingly, REM-CA mutant lose their ability to restrict PVX viral intercellular movement and reduce PD permeability (Gronnier et al., 2017).

**Fig. 4:**
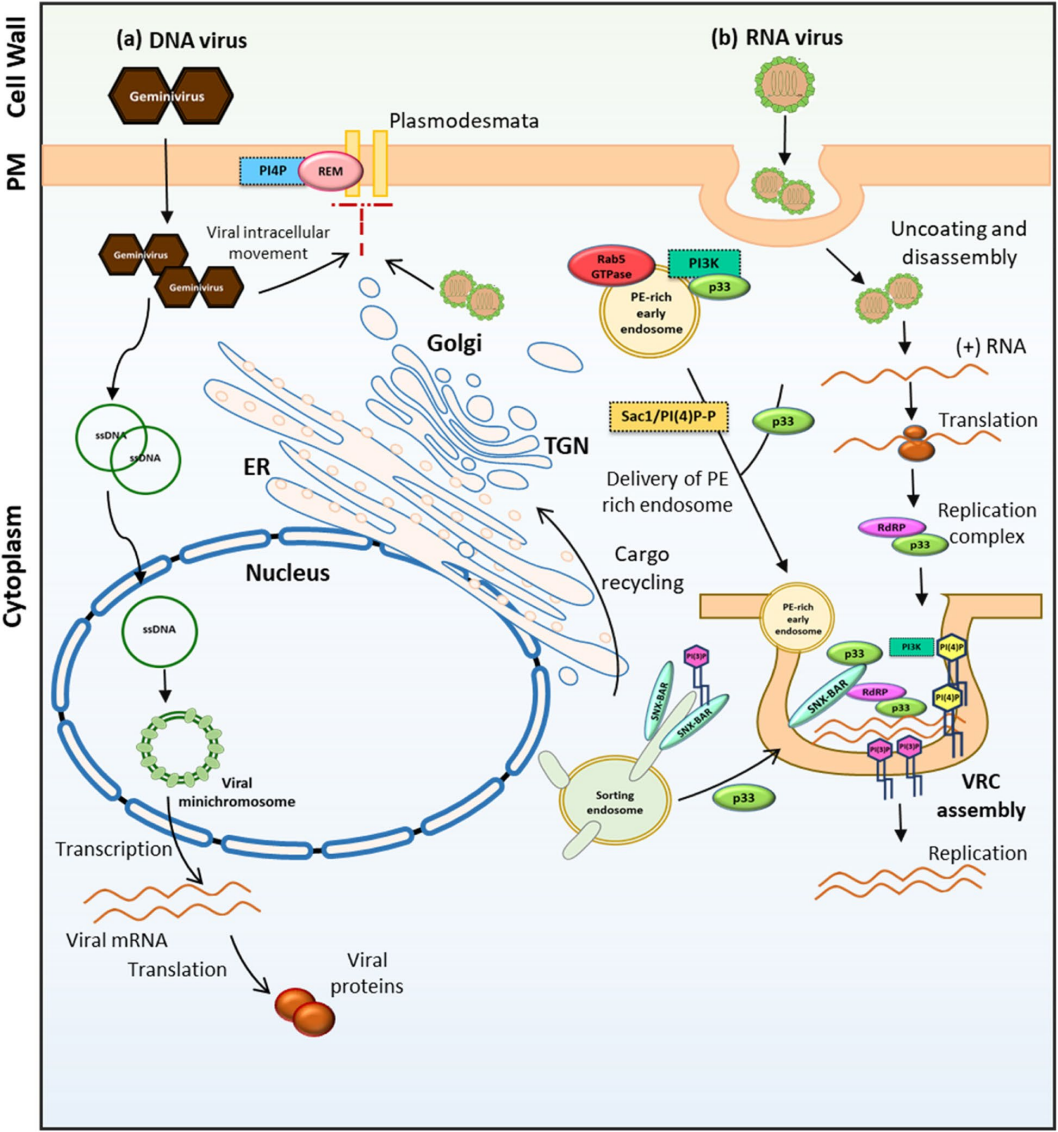
Role of phosphoinositides in virus infection in plants. **a** REMORINs need to localize to the PM and they achieve PM anchoring by interacting with PI(4)P, where they affect plasmodesmata (PD) size exclusion limit and limit viral cell-to cell movement. **b** RNA viruses such TBSV form VRCs for replication. TBSV replication protein p33 helps the recruitment of PE rich Rab5 GTPase-positive endosomes in the TBSV VRC. TBSV p33 replication protein re-localizes the yeast SNX-BAR Vps5p into VRC as a permanent component of the viral replicase complex and the binding Vps5p to PI(3)P is required for this relocalization. PI(3)P, ssDNA, single-stranded DNA; PE, Phosphatidylethanolamine; RdRP, RNA-dependent RNA polymerase, p33*,Tomato bushy stunt virus* (TBSV) replication protein; VRC, viral replicase complexes; SNX-BAR, Sorting nexins-Bin/Amphiphysin/Rvs; PI(3)P phosphatidylinositol-3-phosphate; PI(4)P, phosphatidylinositol-4-phosphate; PI4K, phosphatidylinositol 4- kinase; Rab, Ras-related protein; REM, REMORIN; PM; plasma membrane; ER, endoplasmic reticulum; TGN, *trans*-golgi network

In another example, high-affinity binding between *Rice black streaked dwarf virus* (RBSDV) main capsid protein, P10 and PtdIn(3,5)P_2_ lipid layer was observed using biolayer interferometry (BLI) and subcellular co-localization of PtdIn(3,5)P_2_ and P10 was observed on membranous structures in vector insect cells (Liu et al., 2022). Interestingly, P10 binds and elevates the levels of PtdIn(3,5)P_2_ in the insect vector *Laodelphax striatellus* cell. The virus induced PtdIn(3,5)P_2_ inhibits insect autophagy by preventing the fusion of autophagosomes and lysosomes through activation and helps the virus evade autophagic degradation in the vector (Wang et al., 2022).

### Replication

The crucial role of membrane lipids in the life cycle of human, animal and plant positive-strand RNA is well established. Plant RNA viruses such as *Tobacco mosaic virus* (TMV), *Tomato bushy stunt virus* (TBSV) and *Brome mosaic virus* (BMV) typically harness cellular membranes for replication and viral RNA synthesis (Miller and Krijnse-Locker, 2008; den Boon et al., 2010). These viruses assemble numerous membrane-bound viral replicase complexes (VRCs) with the help of viral replication proteins and host proteins within large viral replication compartments in the cytosol of infected cells. VRCs of many animal and human viruses appear to be enriched in PtdIn and PC (Banerjee et al., 2018; Berger et al., 2011; Hsu et al., 2010; Reiss et al., 2011), whereas many plant and insect vectored animal viruses utilize PE and PC enriched membrane for the same purpose (Xu and Nagy, 2015; Zhang et al., 2016; Zhang et al., 2018). However, the role of PtdIns in plant virus replication is relatively a recent finding. A recent paper (Sasvari et al., 2020) demonstrates the enrichment of PtdIn(4)P within the replication compartment of TBSV as PtdIn(4)P partially co-localized with the TBSV replication protein p33 in yeast cells; reduction in the PtdIn(4)P level due to chemical inhibition in plant protoplasts; or sequestration of free PtdIn(4)P using PtdIn(4)P -binding protein in yeast inhibited TBSV replication (Sasvari et al., 2020). Further, the depletion of two crucial PI4P kinases, Stt4p and Pik1p, also inhibited TBSV replication in yeast cells (Sasvari et al., 2020). In the same study, (Sasvari et al., 2020) dissected the proviral function of Sac1 (suppressor of actin mutations 1-like protein). Sac1 is an ER-localized PtdIn(4)P lipid phosphatase, hence converts PtdIn(4)P to PtdIn and plays a crucial role in virus-induced membrane contact sites (MCSs) formation/function between the ER and peroxisomes, which are required for sterol/PtdIns enrichment within the replication compartment. Sac1 interacts with TBSV replication protein p33 and is present in the MCSs and TBSV replication compartments. Depletion of Sac1 inhibited the recruitment of PE-rich Rab5 GTPase-positive endosomes and the subsequent enrichment of PE in the TBSV VRC. Depletion of Sac1 also negatively affected the recruitment of syntaxin18-like Ufe1p SNARE, which play an important role in TBSV VRC formation (Sasvari et al., 2020, Fig. 4). In another study, using artificial giant unilamellar vesicles (GUVs)-based in-vitro approach to reconstitute TBSV VRCs (Kovalev et al., 2020), showed that PtdIn(3)P, a minor signalling lipid facilitates TBSV replication and incorporation of PtdIn(3)P into the GUV enhanced TBSV replication 2–3 folds (Kovalev et al., 2020). On the same lines, the deletion of Vps34, the PI3K in yeast also reduced TBSV replication (Kovalev et al., 2020). Through interaction with the p33 replication protein, Vps34 is directly recruited into the TBSV VRC and the vesicle transport activity of Vps34 is required for TBSV replication (Feng et al., 2019). In the absence of Vps34, TBSV is unable to successfully recruit PE-rich Rab5-positive early endosomes, which supply PE-rich membranes for TBSV replication compartment membrane synthesis (Feng et al., 2019). In a eukaryotic cell, Sorting nexins (SNXs) are regulators of endosomal sorting. For the SNX-BAR subgroup, a Bin/Amphiphysin/Rvs (BAR) domain is vital for the formation/stabilization of tubular subdomains that mediate cargo recycling (Kovtun et al., 2018). SNXs are recruited to specific subdomains of endosomes by binding to PtdIn(3)P via their Phox-homology (PX) domain and sensing positive membrane curvature through their banana-shaped BAR domains (Chandra et al., 2019). TBSV recruits the SNX-BAR proteins for VRC formation. In yeast, the p33 replication protein of TBSV re-localizes the yeast SNX-BAR Vps5p into VRC as a permanent component of the viral replicase complex and the binding of Vps5p to PtdIn(3)P is required for this relocalization (Feng et al., 2020). The local enrichment of PtdIn(3)P at the VRCs results in positive membrane curvature. SNX-BAR proteins sense, bind and reshape membranes into positive curvature (tube-like forms), which likely stabilizes the narrow neck structure of the VRCs (Feng et al., 2020, Fig. 4). SNX-BAR protein depletion makes the viral double-stranded (ds) RNA replication intermediate ribonuclease-sensitive, confirming the role of SNX-BAR protein and PtdIn(3)P in the stabilization of VRCs (Feng et al., 2020).

The retromer complex plays a role in various cellular processes, including autophagy and lysosome maturation. Tubular transport carriers produced from endosomes recycle cargo goes to the Golgi and ER or to the PM with the aid of the help retromer complex (Cui et al., 2019; Kovtun et al., 2018, Johannes and Wunder, 2016). The core retromer complex consists of three conserved proteins, Vps26, Vps29, and Vps35, which are involved in cargo sorting (Cui et al., 2019; Kovtun et al., 2018, Johannes and Wunder, 2016). TBSV p33 replication protein interacts with the retromer complex, including Vps26, Vps29, and Vps35 (Feng et al., 2021). The TBSV p33-driven retargeting of the retromer into VRCs results in the delivery of important cellular enzymes such as Psd2 (phosphatidylserine decarboxylase), Vps34 (yeast PI3K) and PI4Kα-like to the VRCs enabling the de novo production and enrichment of PE, PtdIn(3)P and PtdIn(4)P within the VRCs (Feng et al., 2021).

### Summary

Emerging findings have greatly expanded our knowledge of the central role PtdIns play in plant-pathogen interaction. The general theme that emerges from these studies suggests the role of PtdIns in pathogen entry, recognition, replication, movement and transmission in the host. However, the data available is preliminary and fragmented, leaving important questions unanswered. The examples discussed in this review emphasize the importance of PtdIns for the pathogen life cycle, however, our current understanding of the precise molecular targets of PtdIn actions is still limited. For instance, numerous proteins with PtdIns-binding domains have been identified, making them prime candidates for regulation. Functional validation of these proteins would help link relevant cellular processes to PtdIns regulation.

Entry of pathogen effector, hyphal growth and defence mechanism such as HR would involve multiple steps of exocytosis and endocytosis. Several studies in plants confirmed the role of PtdIn in vesicle trafficking. For instance, in endocytosis, PI(4,5)P_2_ affects the formation of the clathrin face and in the case of exocytosis, PI(4,5)P_2_ interacts with exocyst subunit SEC3 facilitating the fusion of secretory vesicles (Ischebeck et al., 2013; Bloch et al., 2016).

Further, PI(4,5)P_2_ binding proteins such as Munc-13-1/2 and Sty1 interact with SNARE proteins for assembly at the exocytic site (Martin, 2015). PtdIns have been implicated in the entry of pathogen effector and hyphal growth, but the detailed mechanism involved in PtdIns mediated regulation of endo/exocytosis in response to pathogen attack remains to be studied.

Alteration in levels of PtdIns or PtdIn- pathway enzymes result in a reduction in viral replication (Feng et al., 2019, 2020 Sasvari et al., 2020). It is essential to recall that viruses are obligate cellular parasites and depend on the host machinery for their replication and the discussion in this review on the role of PtdIns in the regulation of nuclear function was added to put into context the emerging pieces of evidence that indicate a direct/indirect interaction of PtdIns with the components DNA replication, transcription and translational machinery (Shoji-Kawaguchi et al., 1995; Ishimaru et al., 2010; Lewis et al., 2011). It would be of special interest to study whether the nuclear function of the PtdIns directly/indirectly affects viral replication. The replication of geminiviruses, a group of plant DNA viruses, is epigenetically regulated by DNA histone methylation. It will be interesting to elucidate the cross-talk between PtdIns and epigenetic regulation of viral replication. Further, future studies are needed to determine the role of PtdIns in plant virus movement and transmission, particularly in the context of REM mediated regulation of PD permeability. Some insight into the function of the PtdIns pathway in vesicle-mediated viral transmission, cytoskeletal re-arrangement, virus movement, and transmission can also be gleaned from animal viruses.

Studies discussed in the above section clearly emphasized the role of PIs in plant-pathogen interactions, however, information on the direct role of PtdIns in regulating defence-responsive genes is limiting, although sufficient evidence exists for such regulation in mammalian systems (Zhang et al., 2019). Defence responses in plants are regulated by plant hormones and PtdIns have also been shown to play a role in SA-dependent defence responses in *A. thaliana* (Antignani et al., 2015). The biological relevance of PtdIn-mediated regulation of plant hormones in plant defence remains to be determined.

Finally, progress in these areas will enable the identification of important candidates and new strategies for crop protection and improvement. Rapidly involving repertoire of cutting-edge microscopy, lipidomics, proteomics and the genetic tool will help shed light on the detailed mechanism coordinating PtdIn-mediated defence response.

## Data Availability

Not applicable.
